# Anisotropic, Vein Width-Tuned Contrast-Limited Adaptive Histogram Equalization Improves Vein Contrast in RGB Images of Forearms

**DOI:** 10.3390/s26206396

**Published:** 2026-10-10

**Authors:** William Lewis, Daniel Cubillos-Rojas, Walfre Franco

**Affiliations:** 1 Department of Dermatology, Beth Israel Deaconess Medical Center, Boston, MA 02215, USA; 2 Department of Dermatology, Harvard Medical School, Boston, MA 02115, USA; 3 Francis College of Engineering, University of Massachusetts, Lowell, MA 01854, USA; walfre_franco@uml.edu; 4 Department of Dermatology, University of Massachusetts Chan Medical School, Worcester, MA 01655, USA

**Keywords:** vein, venous, smartphone, RGB, CLAHE, contrast enhancement, image processing, vein imaging

## Abstract

Locating subcutaneous veins for phlebotomy and intravenous cannulation is a common clinical task; vein finders, including near-infrared or RGB-based imagers, are increasingly used to aid this process. Image processing pipelines used to enhance vein images often employ a contrast enhancement step, such as contrast-limited adaptive histogram equalization (CLAHE) applied over a grid of tiles independent of vein size or orientation. We hypothesized that matching CLAHE tile geometry and orientation to the expected orientation and geometry of the forearm and its veins could improve vein contrast. 18 RGB images of the volar forearm were processed by a venous spectral index (R-G) and enhanced by CLAHE across a matrix of 121 possible tile widths and heights as a function of vein width. Weber contrast in each image was computed over line profiles drawn across vein regions. The contrast-maximizing tile was taller than wide in 89% of images (median height-to-width ratio 3.0; *p* = 0.001), and the best tall tile (3W × 10W) produced higher contrast than the same tile rotated by 90° in 83% of images (*p* = 0.004). Matching CLAHE tile dimensions to forearm and vein geometry may improve image processing pipelines for vein visualization.

## 1. Introduction

The identification of veins is an important part of medical care in a variety of settings, including pre-hospital settings, outpatient clinics, and inpatient medical care. Identifying veins by visual inspection alone can be challenging, particularly in patients with darker skin pigmentation, low intravascular volume, small veins (e.g., pediatric patients), or patients with excess subcutaneous fat [1]. As a result, a variety of tools exist to assist with the visualization and identification of veins including near-infrared (NIR) vein finders, illumination sources, glasses with colored lenses, and ultrasound imaging devices, among others [2]. These tools range in cost from relatively inexpensive (illumination sources, glasses) to expensive and bulky (ultrasound) and require varying degrees of training. In general, the clinical aim of vein-finding devices is the identification of veins, especially in the forearm or antecubital fossa, that may be used for administration of intravenous products or venipuncture for blood draws, in patients where palpation and visual inspection are unrevealing. Both near infrared vein imaging devices [3] and point-of-care ultrasound [4] have shown benefit for reducing intravenous cannulation procedure time and number of cannulation attempts.

Although ultrasound imaging is capable of producing perhaps the most high-quality images of subcutaneous veins for clinical settings, use of ultrasound for venous cannulation requires significant training in image acquisition, image interpretation (as the images are cross-sectional and reflect tissue echogenicity), and simultaneous venipuncture while performing imaging. Additionally, ultrasound machines are bulky, expensive, and unlikely to be available outside of a hospital setting. NIR and RGB imaging methods are far more easily interpretable by a clinician (e.g., nurse, phlebotomist, physician or EMT) as they display their image data in a format akin to standard visual inspection. A variety of FDA-cleared NIR vein imaging devices exist for use in clinical settings. RGB vein imaging has been used for research purposes [5] and may provide a low-cost alternative.

Standard consumer cameras such as smartphone cameras can be used to generate enhanced images of subcutaneous veins by a variety of methods [6,7,8]. One such method is the use of a red-green-blue (RGB) venous spectral index (VSI), obtained by subtracting the green channel of the image from the red channel (R-G) [7]. The VSI reduces noise from the red channel image (which contains the relevant venous deoxyhemoglobin absorption signal) by subtracting superficially scattered light and specular reflection, which are present as additive noise across both the red and green channels. Another approach to vein visualization in standard color camera images is Wiener spectral estimation [6], which may be combined with bilateral filtering to adjust for spatial variations in illumination [8]. All RGB camera-based approaches to vein visualization are sensitive to the spectrum of the illumination source, as red channel deoxyhemoglobin absorption provides the contrast for veins against background tissue [8]. The outputs from Wiener spectral estimation or application of a spectral index are generally low-contrast grayscale images that benefit from further contrast enhancement or filtering for optimal visualization [6]. NIR imaging modalities use custom illumination sources and detectors to visualize the greater contrast between oxy- and deoxyhemoglobin in the 740–940 nm wavelength range [9].

The outputs of vein imaging tools, whether acquired by NIR or RGB imaging, are generally produced by similar pipelines: an image in which veins appear either darker or brighter than background skin is obtained, its contrast is enhanced, and a vein map is produced by some combination of thresholding, vesselness filtering, morphological operations and/or use of neural networks or machine learning. The contrast enhancement steps utilized in these pipelines affect the downstream output, and faint veins that are not adequately separated from background skin at this step may not be recovered later. Global histogram equalization or Contrast-limited adaptive histogram equalization (CLAHE) are used as a contrast enhancement step in several near infrared vein image enhancement pipelines [10,11,12].

Global histogram equalization and standard CLAHE are isotropic. CLAHE works by dividing the image into regions called tiles, performing histogram equalization within each tile, clipping each tile’s histogram at a preset limit to avoid noise amplification, and bilinearly interpolating between neighboring tile mappings to suppress tile-boundary artifacts [13,14]. CLAHE tiles much smaller than the feature of interest in an image tend not to enhance feature characteristics, whereas very large tiles simply tend to approach global histogram equalization in an image [15]. In standard CLAHE, tile size is set by the image size and framing, and is independent of the image contents. Tile size relative to the size of a vein in an image will vary with resolution and working distance, meaning that a tile setting appropriate for one image will not necessarily be optimal for another. Additionally, on a forearm, tiles near the limb margin contain background scenery as well as skin and vein, so the local tile histogram is stretched to include background and the part of the output range available to separate skin and vein is thereby reduced. Importantly, in images of the volar forearm, this is where clinically-relevant structures like the basilic and cephalic veins are expected to lie. Because CLAHE is already used for vein image processing pipelines that acquire images under these conditions [11] it is worth exploring whether it might be further optimized.

Anisotropic image processing methods have shown promise for vein enhancement in near-infrared vein images; after near-infrared image acquisition, Gabor filtering has been applied for palm vein recognition [16], and Gabor and Retinex filtering using a fuzzy-based fusion method [17] has been applied for the enhancement of near-infrared images of finger veins. Because forearms and forearm veins have a characteristic geometry and directionality, we hypothesized that matching CLAHE tile size, shape and orientation to the expected orientation and geometry of the forearm and its veins could improve vein contrast compared with standard CLAHE.

## 2. Materials and Methods

### 2.1. Image Preprocessing and Vein Measurement

18 digital color images of the forearm were manually rotated so that the long axis of the forearm aligned with the vertical axis of the image and cropped to a region of interest over the forearm using the Matlab Image Processing Toolkit (Matlab R2024a, Mathworks, Natick, MA, USA). The 18 images were of 18 different adults from public repositories (Flickr via Openverse and Wikimedia Commons); age, gender and skin phototype were not recorded, although the images included a range of apparent skin tones. All images were 8-bit-per-channel JPEG files (0.7 to 20.7 megapixels); after rotation and cropping the analyzed regions ranged from 153 × 455 to 1596 × 3208 pixels.

**Figure 1:**
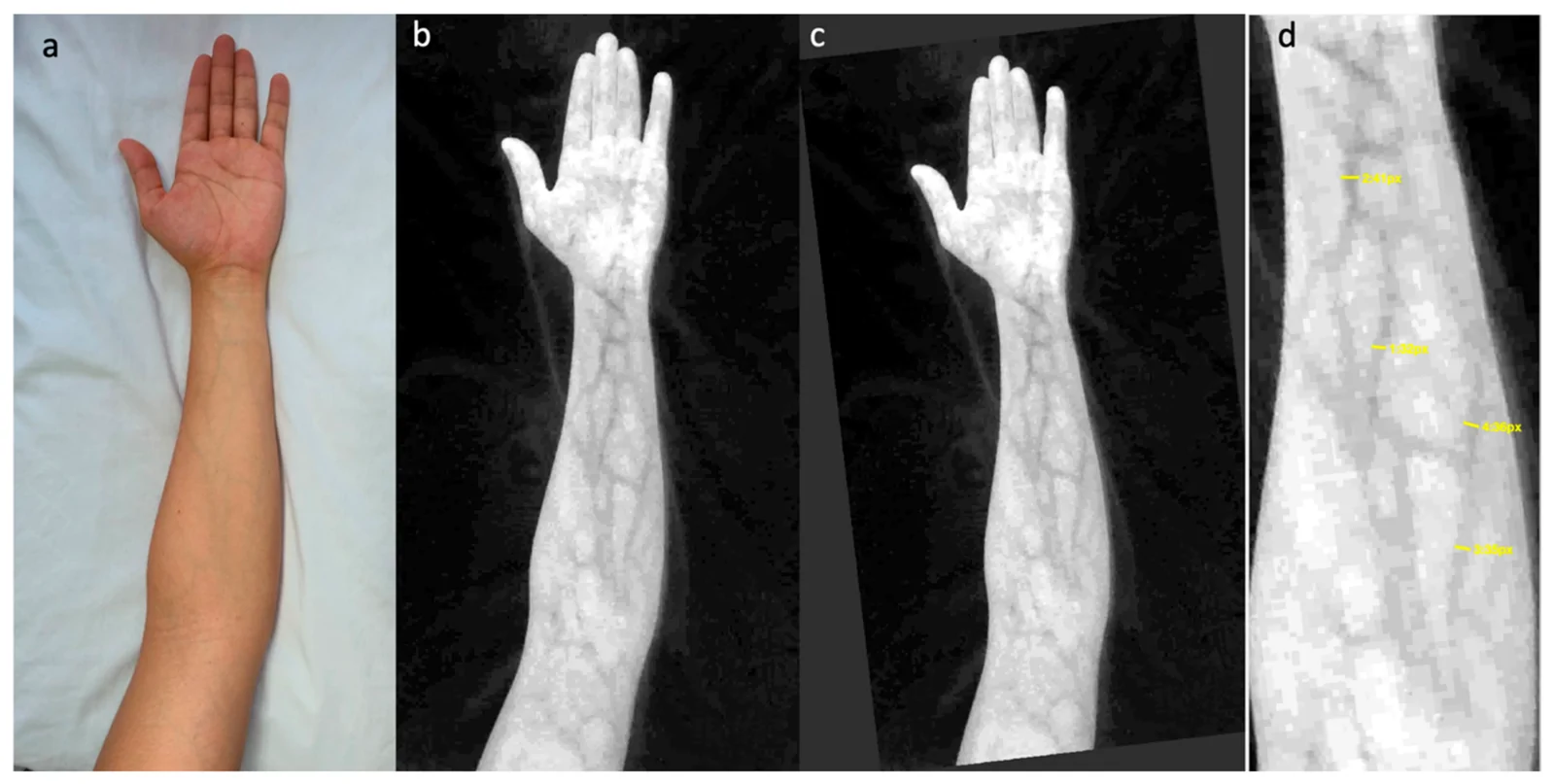
Workflow for image processing: Images of forearms (**a**) were processed in Matlab Image Processing Toolbox using the Venous Spectral Index, red minus green (**b**). Images were then manually rotated to align the axis of the forearm and veins approximately with the vertical axis of the image (**c**). Four vein cross sections were then manually delineated using the drawline function in matlab (**d**). Yellow lines in (**d**) display the vein cross-sections; adjacent yellow text indicates the line segment’s label and its length in pixels.

Images were then processed using the VSI, red minus green (R-G). After processing, the mean vein width W, in pixels, was recorded from four manually-drawn vein cross-sections per image using the drawline function in Matlab (Figure 1). W was the apparent edge-to-edge vein width judged by a clinician on the VSI image; four sites distributed along the visible veins were used to average local variation in caliber, and the same four sites were used for calculating vein contrast. Mean vein width W ranged from 7.6 to 69.7 pixels.

### 2.2. Contrast Enhancement

**Figure 2:**
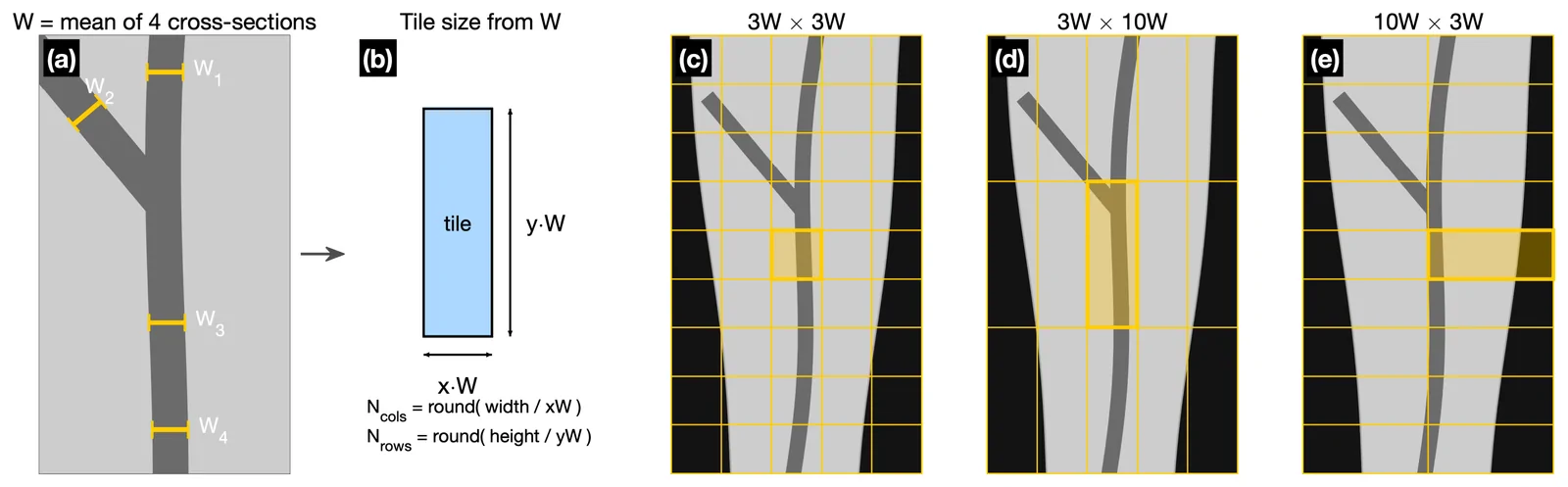
Method for vein width-matched CLAHE implementation. (**a**) The vein width W is taken as the mean of four manually drawn cross sections on the enhanced image. (**b**) Tile width and height are specified in terms of vein width and, together with the dimensions of the image, determine the number of tiles passed to the adapthisteq function in Matlab. An example of resulting tile grids, with one tile highlighted, for (**c**) a 3W × 3W tile (**d**) a 3W × 10W tile and (**e**) a 10W × 3W tile are shown.

Each image was enhanced by CLAHE using the adapthisteq function in Matlab across an 11 by 11 matrix of possible tile widths and heights as a function of vein width W (Figure 2). Conventional CLAHE uses square tiles of fixed number (for example, 8 × 8) which are dependent upon the dimensions of the input image but independent of the image contents or the desired features to be enhanced; here, the tile width and height were set to multiples (0.5, 1, 1.5, 2, 2.5, 3, 4, 5, 6, 8 and 10) of the measured apparent vein width and height. The adapthisteq function in Matlab takes, as an argument, a number of tiles (NumTiles) in rows and columns. In order to map the tile dimensions and number of tiles in the image to the vein width, for a given tile width xW and height yW, the number of tiles in the image was set to round(imagewidth/xW) horizontally and round(image height/yW) vertically, with a minimum of 2 per dimension. Conditions with y > x are tall tiles elongated along the forearm axis, conditions with y < x are wide tiles elongated across the forearm, and conditions with y = x are square tiles. Other input arguments to the adapthisteq function include ClipLimit (the contrast enhancement limit) and NBins (the number of histogram bins used to build a contrast enhancing transformation); these were set at 0.05 and 256 respectively for all image contrast transformations. In total, 2178 CLAHE-enhanced images were generated (18 original images × 121 tile geometries).

### 2.3. Contrast Measurement

After application of CLAHE, a line intensity profile running from normal skin through the vein and onto normal skin on the other side was drawn, with total length 3W (three times the vein width at that location in the image). Vein intensity *V* was calculated as the mean intensity of the central 15% of the line profile, and skin intensity *S* was calculated as the mean intensity of the outermost 15% of each end of the line profile. Weber contrast *C* was calculated by the equation:

(1) $$C=\frac{\left(S-V\right)}{S}$$

For each image, *C* was computed and averaged across the four vein regions for every CLAHE condition (a total of 8712 vein contrast measurements). Measurements were then normalized across all 121 CLAHE conditions per image, with the highest-performing condition set at a value of 1.

### 2.4. Statistical Analysis

The study was a within-image, repeated-measures design; the primary outcome was the aspect ratio of the contrast-maximizing tile in each image and the secondary outcome the paired comparison of the best tall tile with the same tile rotated by 90°. For each image, the tile aspect ratio (height/width) that gave the highest contrast was identified, and a two-sided sign test tested whether the highest-contrast aspect ratios were >1 (taller than wide). A Wilcoxon signed-rank test then compared, within each image, the contrast of the best tall tile against that of a wide tile (the same tile geometry rotated 90 degrees).

### 2.5. Pipeline Demonstration

To illustrate the effect of tile geometry on a downstream vein-detection step, one image (vein width W = 50 px; cropped image 1597 × 3185 px) was processed by VSI followed by CLAHE using vein-tuned anisotropic tiles (3W × 10W) versus square tiles. Results underwent subsequent global thresholding using Otsu’s method via the graythresh function in Matlab. The two vein masks were compared visually.

## 3. Results

Optimal CLAHE tile geometry was consistently anisotropic along the axis of the forearm (Figure 2). Optimal tile width had a median of 3W and optimal tile height had a median of 8W; the median optimal aspect ratio was 3.0 (interquartile range 1.5–3.3). The median Weber contrast was 0.357 with the optimal 3W × 10W tall tile versus 0.329 for the corresponding 10W × 3W wide tile.

**Figure 3:**
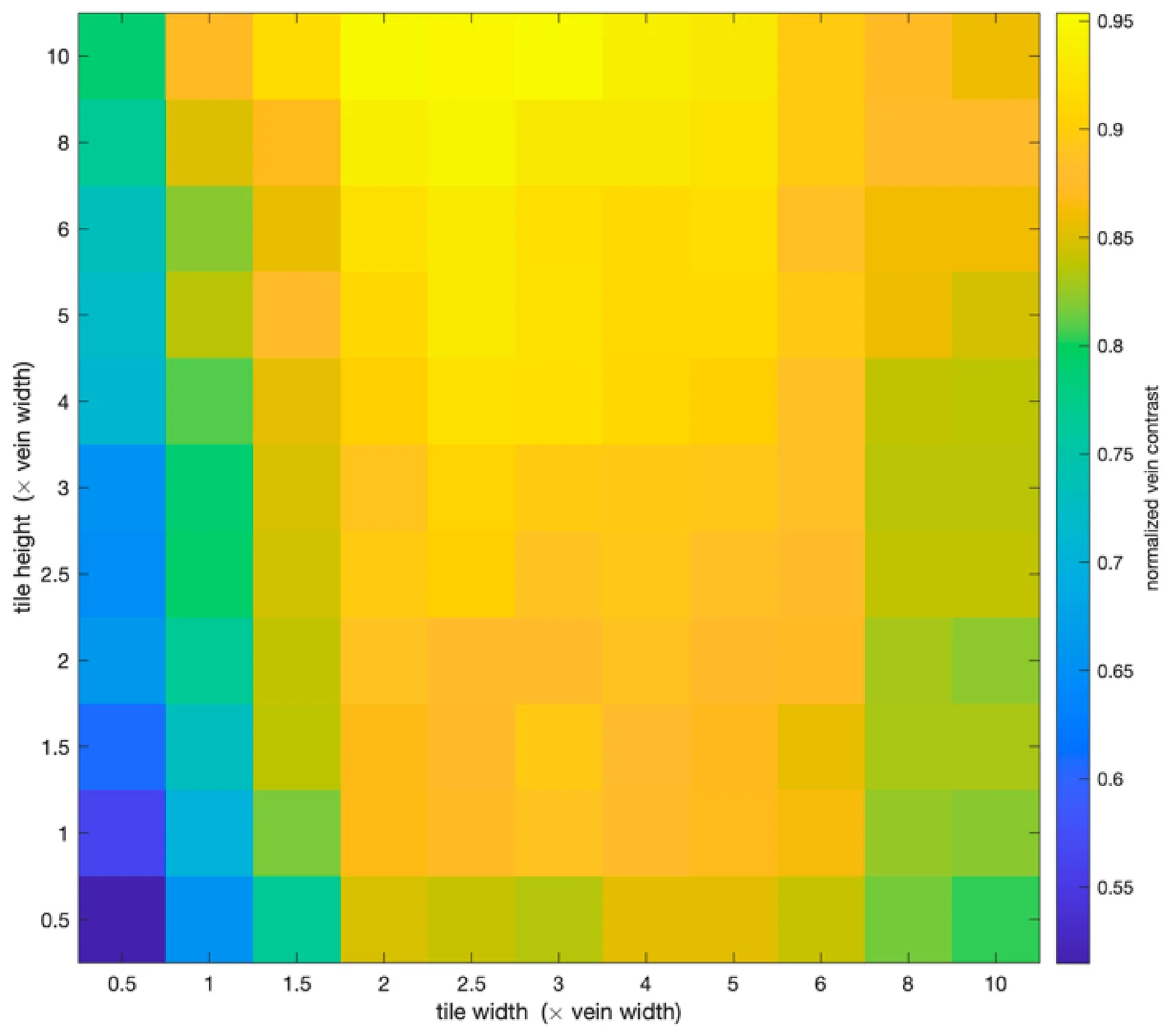
Median normalized Weber contrast along the vein line profile as a function of CLAHE tile geometry. Tile width (*x*-axis) and height (*y*-axis) are given in units of vein width W. Brighter values indicate higher vein-skin contrast. In general, narrow and tall tile geometries produced higher contrast than square or wide geometries, with the highest median contrast seen for tiles that were 3W wide and 10W tall.

**Figure 4:**
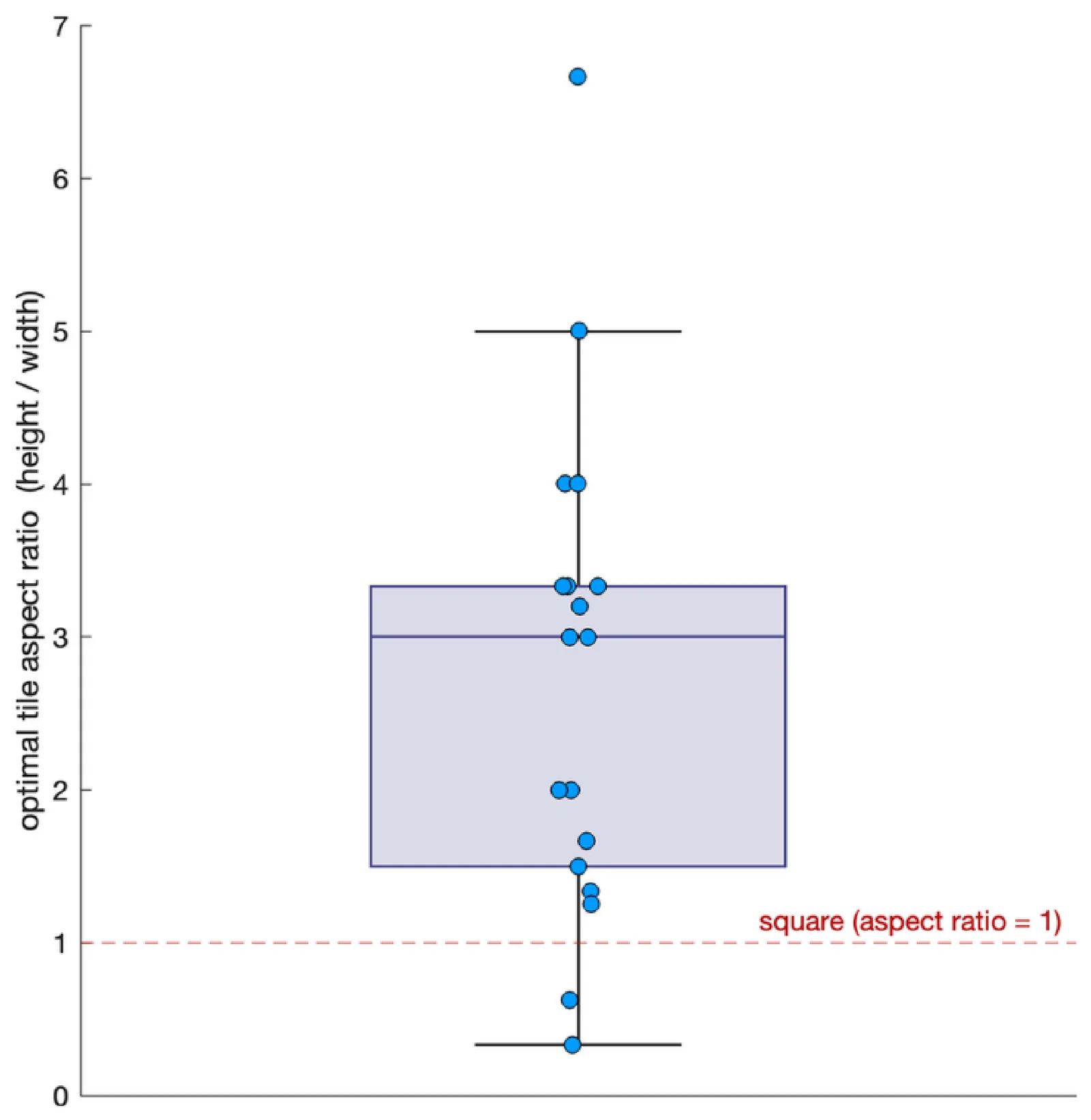
Optimal tile aspect ratio (height/width) for each of the 18 images. Each point reflects one image’s highest-contrast tile aspect ratio. The box indicates the median and the interquartile range. Values above the dashed line indicate tiles elongated along the axis of the vein/forearm. The dashed line corresponds with an aspect ratio of 1, i.e., a square tile.

CLAHE performed with square tiles corresponds to the diagonal from bottom left to top right in Figure 3; the median normalized contrast was 0.95 for the tall 3W × 10W tile, 0.90 for the square 3W × 3W tile and 0.84 for the wide 10W × 3W tile. Optimal CLAHE tile aspect ratios for each image are shown in Figure 4.

**Figure 5:**
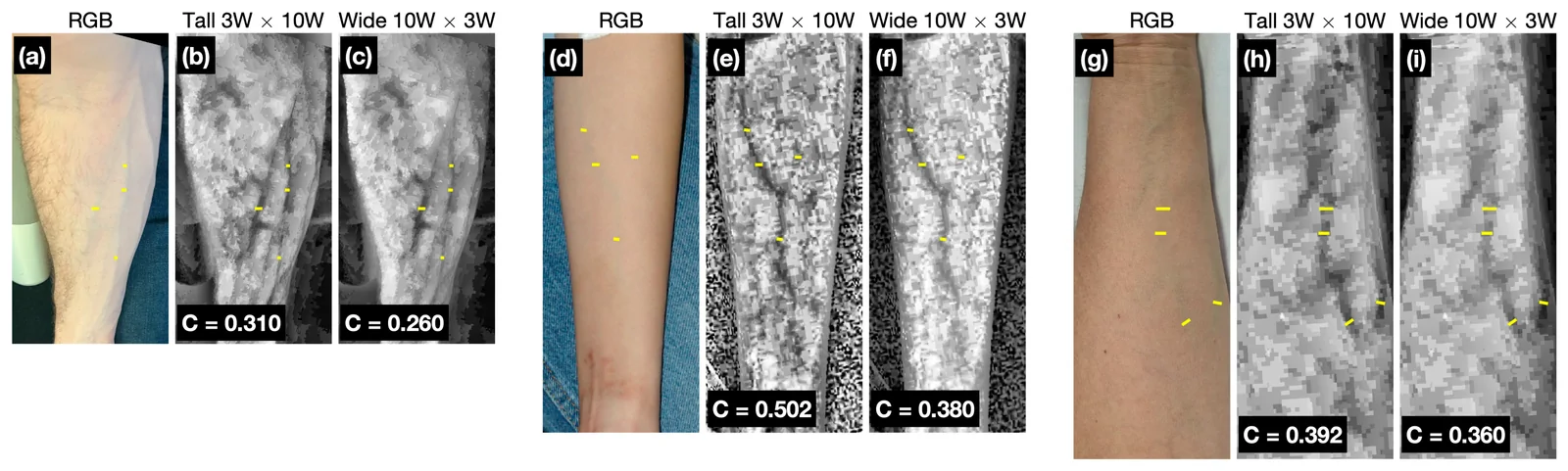
Representative RGB forearm images (**a**,**d**,**g**) and their corresponding VSI images with tall (**b**,**e**,**h**) versus wide (**c**,**f**,**i**) CLAHE tiles. Yellow horizontal lines across the 4 vein segments show the sites chosen for line profile measurements. Weber contrast C is annotated on each image. Tall tiles rendered veins more sharply and showed improved skin-vein contrast across the line profiles.

Within each image, the best tall tile produced higher contrast than the same tile rotated 90° (wide) in 83% of images (Wilcoxon signed-rank *p* = 0.004). Examples of the best-performing tile height-to-width ratio and its inverse applied to images of veins are shown in Figure 5.

**Figure 6:**
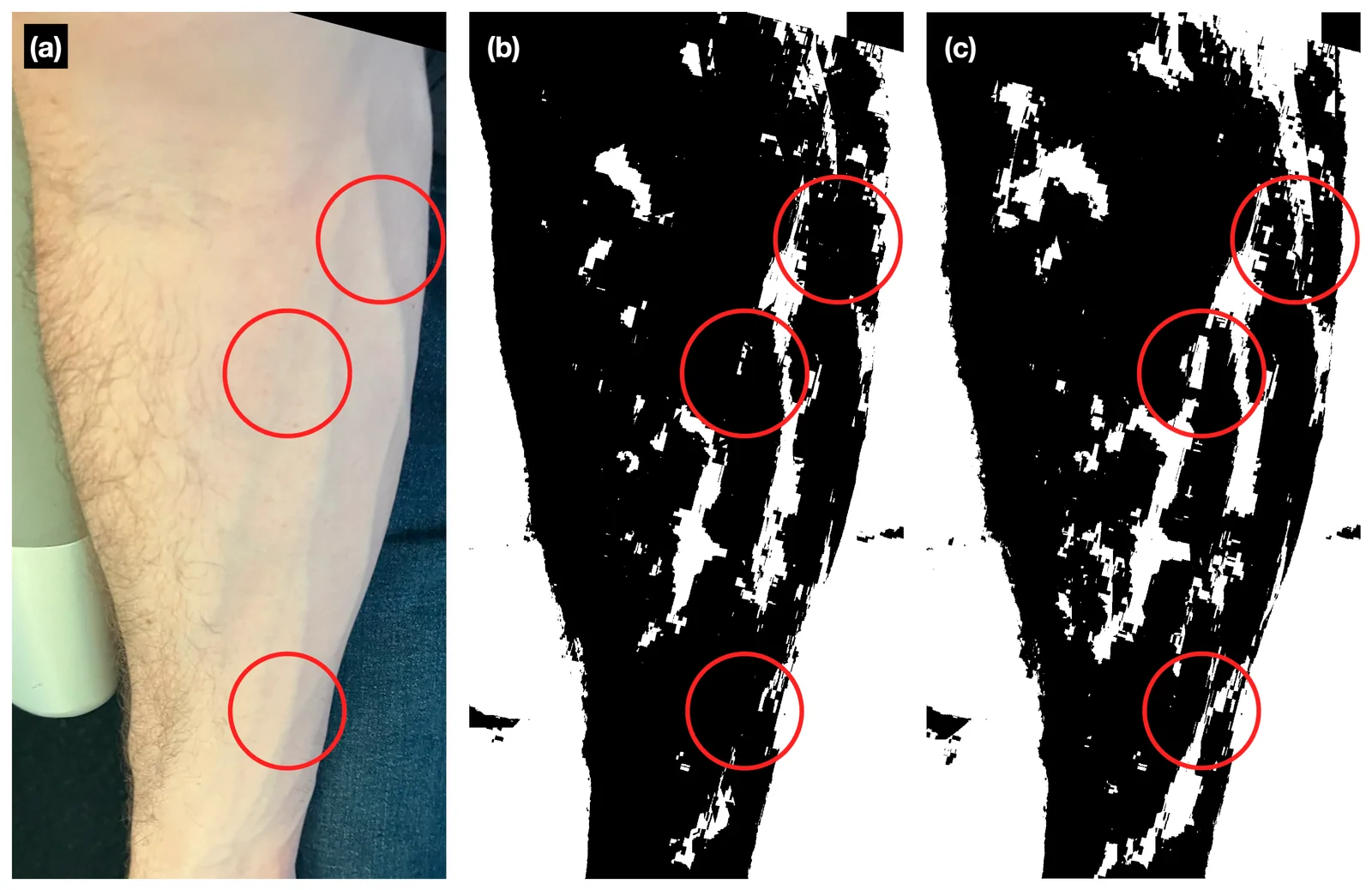
Effect of CLAHE tile geometry on a vein segmentation pipeline consisting of application of VSI, followed by isotropic versus anisotropic CLAHE, followed by masking using Otsu’s method via the graythresh function in Matlab. (**a**) RGB photograph of the volar forearm. (**b**) Vein mask obtained by applying Otsu’s global threshold to the VSI image after CLAHE with square tiles and (**c**) after CLAHE with the vein width-tuned tile of 3W × 10W; all other CLAHE parameters were identical. Red circles mark the same three regions in each panel, and correspond to regions of the median and basilic veins of the forearm that are missed or discontinuous in (**b**) and highlighted in (**c**).

To demonstrate effects of tile geometry on the output of a vein-masking pipeline, global thresholding of the CLAHE output for an example image with a square 6W × 6W tile versus a 3W × 10W tile was performed using the graythresh function in Matlab. Using standard CLAHE, the veins were segmented as a series of disconnected fragments, whereas with the tile elongated along the forearm, veins were segmented as near-continuous structures (circled regions in Figure 6).

## 4. Discussion

In this study of digital images of forearm veins, we found that Weber contrast between the relevant portions of a line profile extending across a vein could be improved by applying CLAHE with anisotropic tile configurations; specifically we found that the best-performing tile configurations extended along the axis of the forearm and had width nominally 3 times the width of the forearm veins. CLAHE tiles that were elongated parallel to the forearm’s long axis improved our contrast measure compared with long tiles oriented perpendicular to the forearm’s long axis, and CLAHE tiles smaller than one vein width or wider than about 6 vein widths did not enhance contrast as well.

In the images studied, background non-skin regions remained after cropping, meaning that the anisotropic contrast improvement effect we observed may be partly due to CLAHE tiles’ configuration and orientation relative to non-skin background that is likely to take on more extreme VSI intensity values compared with skin. CLAHE tiles which include skin, vein, and background potentially limit the dynamic range available to vein and skin, whereas tiles containing only skin and vein can stretch the contrast between these elements more effectively. This impact is relevant to practical application; the naturally tapered geometry of forearms means that images captured under typical clinical conditions that include the entire forearm on a standard image sensor will also likely include a background region, and both the cephalic and basilic veins are likely to be found close to the edges (i.e., the forearm-background boundary) of a standard image of the volar forearm. Our findings regarding tile size (that the optimal tile width is about 3 times the diameter of the vein) are consistent with prior work on CLAHE suggesting that optimal enhancement of image features occurs at tile diameters slightly larger than the feature of interest [13,15].

A variety of tools (e.g., OpenCV libraries, Apple Vision frameworks) exist for smartphone-based image segmentation and analysis of human features such as body parts in images, which may be applied live at the time of video or image capture on a smartphone or similar device. Because forearm veins may be expected to have a relatively narrow range of possible widths relative to forearm width, such tools could potentially enable application of anisotropic, feature-specific CLAHE for vein visualization in live video at time of capture, even when veins are not directly visualized or measured (i.e., by measurement of forearm geometry and orientation). When advanced image analysis tools are unavailable, simply adjusting the capture process to position the forearm in the appropriate position or at the appropriate distance from the camera lens may potentially suffice to optimize vein visualization using the method described in this paper.

Limitations of this study included the use of compressed publicly-available images which resulted in some image artifacts (banding and blocks of homogeneous intensity within images). Due to availability of public-domain images, the sample size, limited to the volar forearm, was modest. Application of image processing at the time of capture or on RAW images may circumvent issues with image compression. Age, gender and skin phototype of the individuals were not available for these images; darker phototypes, in which enhancement is of greatest clinical interest, warrant deliberate representation in future work. No independent reference standard for vein location was available; vein locations in images were identified by a clinician. The results therefore describe relative vein-skin contrast at sites where a vein was already visible rather than detection of otherwise invisible veins. Finally, although we examined a range of CLAHE tile sizes relative to vein width, it is possible that optimal geometries may lie further outside this range; future studies could explore a broader range of CLAHE tile geometries.

## 5. Conclusions

In RGB digital images of human forearms to which a venous spectral index has been applied, CLAHE tiles about three vein widths wide and elongated along the long axis of the forearm produced higher vein-skin contrast than square tiles of the same width or the same tiles oriented across the forearm. Use of anisotropic CLAHE tile geometry with size adjusted based on vein diameter may improve consumer digital camera-based vein image enhancement.

## Data Availability

Data is available from the corresponding author upon reasonable request.

## Abbreviations

The following abbreviations are used in this manuscript:

VSI	Venous Spectral Index
RGB	Red Green Blue
NIR	Near Infrared
CLAHE	Contrast-Limited Adaptive Histogram Equalization
